# Optical Devices for the Diagnosis and Management of Spinal Cord Injuries: A Review

**DOI:** 10.3390/bios14060296

**Published:** 2024-06-05

**Authors:** Sonika Sharma, Neeti Kalyani, Taposhree Dutta, Jesús Salvador Velázquez-González, Ignacio Llamas-Garro, Bora Ung, Joan Bas, Rakesh Dubey, Satyendra K. Mishra

**Affiliations:** 1 Department of Physics, Graphic Era Hill University, Dehradun 248002, Uttarakhand, India; sharma.s8529@gmail.com; 2 Department of Biotechnology and Biomedicine, Denmark Technical University, 2800 Kongens Lyngby, Denmark; neeka@dtu.dk; 3 Department of Chemistry, Indian Institute of Engineering Science and Technology, Shibpur, Howarh 711103, West Bengal, India; taposhreedutta812@gmail.com; 4 Navigation and Positioning, Center Technologic de Telecomunicacions de Catalunya (CTTC), Avinguda Carl Friedrich Gauss, 11, 08860 Castelldefels, Spain; jesus.salvador.velazquez@cttc.es (J.S.V.-G.); illamas@cttc.es (I.L.-G.); 5 Electrical Engineering Department, Ecole de Technologie Superieure, Montreal, QC H3C 1K3, Canada; bora.ung@etsmtl.ca; 6 Space and Resilient Communications and Systems (SRCOM), Center Technologic de Telecomunicacions de Catalunya (CTTC), Avinguda Carl Friedrich Gauss, 11, 08860 Castelldefels, Spain; joan.bas@cttc.es; 7 Institute of Physics, University of Szczecin, 70-453 Szczecin, Poland; rakesh.dubey@usz.edu.pl

**Keywords:** spinal cord, spinal cord injury, optical coherence tomography, fluorescence imaging, photoacoustic imaging, plasmonic nanoparticles, wearable optical technology, neuroimaging, fiber Bragg grating

## Abstract

Throughout the central nervous system, the spinal cord plays a very important role, namely, transmitting sensory and motor information inwardly so that it can be processed by the brain. There are many different ways this structure can be damaged, such as through traumatic injury or surgery, such as scoliosis correction, for instance. Consequently, damage may be caused to the nervous system as a result of this. There is no doubt that optical devices such as microscopes and cameras can have a significant impact on research, diagnosis, and treatment planning for patients with spinal cord injuries (SCIs). Additionally, these technologies contribute a great deal to our understanding of these injuries, and they are also essential in enhancing the quality of life of individuals with spinal cord injuries. Through increasingly powerful, accurate, and minimally invasive technologies that have been developed over the last decade or so, several new optical devices have been introduced that are capable of improving the accuracy of SCI diagnosis and treatment and promoting a better quality of life after surgery. We aim in this paper to present a timely overview of the various research fields that have been conducted on optical devices that can be used to diagnose spinal cord injuries as well as to manage the associated health complications that affected individuals may experience.

## 1. Introduction

The spine’s health is of utmost importance for the proper function of the human body. The spine protects the spinal cord, a column of nerves connecting the brain with the rest of the body. The spine is made up of vertebrae, which enclose the spinal cord. The spinal cord is a tubular structure running from the base of the brain down to the lower back, encased within the vertebral column. Comprising many nerve fibers, it features ascending tracts that transmit sensory information from the body to the brain and descending tracts that relay motor commands to muscles. The spinal cord comprises grey and white matter, and the share of each case in spinal cord composition is 40 and 60%, respectively. Grey matter consists of nerve cells responsible for muscle movement, sensory information, vibrations, and more. The surrounding white matter consists of nerve fibers that transmit signals between the brain and the rest of the body and hence are fundamental for coordination and movement. Cerebrospinal fluid (CSF) is a jelly-like substance that surrounds the spinal cord and protects the nerve fibers and nerve cells. Having mentioned the functioning of all spinal cord constituents, it is straightforward to understand why spinal health is vital for healthy human life [1]. Based on the source of injury, spinal cord injuries (SCIs) can be divided into traumatic and non-traumatic. Traumatic SCI often stems from external physical accidents like car crashes, falls, sports-related incidents, or acts of violence. These events may lead to fractures, dislocations, or spinal cord compressions. On the other hand, non-traumatic SCI occurs due to the progression of existing acute or chronic illnesses such as tumors, infections, spinal cord ischemia (resulting from insufficient blood flow), or degenerative diseases like multiple sclerosis [2].

After a spinal cord injury, individuals often face a multitude of underlying problems that extend beyond the immediate physical impairments. These issues encompass secondary complications like muscle atrophy, pressure ulcers, urinary tract infections, and respiratory problems. Additionally, there are profound emotional and psychological challenges, including depression, anxiety, and adjustment disorders, as affected individuals adapt to their changed circumstances [3,4]. Spinal cord injuries impose severe physical, social, and occupational burdens on patients and their families. These injuries lead to a loss of independence and a persistent increase in mortality rates throughout the affected individuals’ lives. Individuals who have suffered a spinal cord injury face a significantly higher risk of premature mortality, ranging from two to five times greater than those without such damage. This discrepancy in survival rates is more pronounced in low- and middle-income countries [5]. Direct costs associated with caring for SCI patients range from USD 1.2 to 5.1 million per patient over their lifetime [6], underscoring prevention’s importance as the primary intervention strategy for SCIs. However, in cases where prevention is not possible, developing effective treatments becomes critically important. Annually, an estimated 250,000 to 500,000 individuals suffer from spinal cord injuries globally. The incidence of SCI varies globally, with notable differences among developed regions. In 2019, there were 0.9 million incident cases of SCI globally. In North America, the incidences of traumatic SCI were higher, with 0.11 million cases compared with 0.107 and 0.047 million patients in Europe and Southeast Asia, respectively. This discrepancy is attributed to higher rates of violent crime and self-harm in North America [7].

No cure exists for SCI, but research endeavors are making significant strides in multiple domains. Ongoing research is exploring promising avenues such as stem cell the-rapy, neuroprotective drugs, and electrical stimulation to improve the functional outcomes for individuals with SCI. Stem cell therapy holds potential for tissue regeneration [8,9], while neuroprotective agents aim to minimize secondary damage [10]. Electrical stimulation techniques are also being developed to enhance motor function and improve the quality of life for those with SCI [11,12,13]. Cellular therapies, molecular therapies, and therapies addressing myelin inhibitors and extracellular matrix modifiers show potential, with some advancing toward clinical trials [14,15]. While some of these interventions are experimental, others, such as nerve growth factor administration for Alzheimer’s disease, have entered clinical trials [16]. While these advancements are promising, it is essential to understand that comprehensive rehabilitation and supportive care remain crucial for managing the consequences of SCI and improving the overall well-being of affected individuals. Rehabilitation is pivotal in enhancing locomotor function after SCI, harnessing the spinal circuitry’s inherent plasticity and functionality below the lesion site. However, challenges persist in determining the safety and efficacy of these therapies for human SCI [17].

Based on the severity and the extent of sensory and motor function impairment, spinal cord injuries can be classified into complete SCI and incomplete SCI. In cases of complete SCI, there is a total loss of sensory and motor function below the injury site, resulting in a complete disconnection between the brain and the affected body parts. Incomplete SCI occurs when some sensory or motor function remains below the injury site; it encompasses a range of conditions where partial communication between the brain and the body persists. The specific deficits can vary significantly [18].

The precise diagnosis of these spinal cord injuries is crucial, and for this purpose, imaging technologies play a significant part. Through imaging techniques, aid for correctly navigating surgical tools, including inspecting needle probes, can be achieved, which is a critical aspect of such sophisticated surgical procedures [19]. In spinal surgery, complex anatomy, the deformation of the outer layers of the spinal cord, or after-effects of the injury may block the advancement of the inspecting probe, which can complicate the surgical procedure and enhance the risk of fatal consequences. Traditionally, to address such a problem, a spinal endoscopy (epiduroscopy) approach involved the intensive use of continuous X-rays for scanning, exposing the patient to harmful radiation [20,21,22]. The well-established radiation-less in vivo imaging techniques for spinal cord imaging in living beings (i.e., animals and humans) are magnetic resonance imaging (MRI), ultrasound, and diffusion tensor imaging (DTI) [23,24,25,26]. These commonly used imaging techniques provide in vivo serial imaging of the spinal cord in the preclinical stage but suffer from substandard image resolution, which hinders the effective visualization of requisite microscale anatomical structures such as the circulatory system and neural pathways. Not only this, but these imaging techniques also struggle with poor tissue specificity, hence requiring an extrinsic dye to distinguish between the circulatory system and solid tissue structures. Furthermore, CT and MRI imaging are expensive and cumbersome and require compatibility adjustment of the surgical tools with an imaging procedure. However, the complex anatomy of the spinal cord and surrounding structures causes a hindrance in accessibility to the spinal cord for human research as the critical aspect in such a sophisticated surgical procedure is to achieve the correct navigation of the surgical tools [27].

Various optical imaging techniques contribute to the guidance imaging of spinal interventional procedures [28]. They are extensively used for the precision investigation of irregularities in spine structure due to traumatic injuries, with fewer complications [29]. Traditionally, fluoroscopy, computed tomography, and reflectance spectroscopy techniques have been used for guided imaging. Despite their extensive use, these techniques come with individual shortcomings. Despite its low cost and ease of use, fluoroscopy is unable to detect soft tissues like blood vessels and nerves, which can lead to surgical errors [30]. Anatomical resolution and needle placement accuracy are improved through CT fluoroscopy. It cannot, however, identify the positioning of a needle in a tiny radicular or medullary vein and unfortunately, because of its low spatial resolution (~1 mm), it cannot identify the needle’s insertion into smaller radicular or medullary arteries. This method is not only laborious, but it is not appropriate for everyday tasks [31,32]. Further use of reflectance spectroscopy resolves the issue regarding image resolution in the abovementioned techniques but provides only 1-D analysis, which lacks intuitive information for guidance.

This context makes optical imaging techniques more advantageous than conventional imaging techniques. Through improving image resolution, monitoring in real time, and avoiding invasive procedures, optical imaging can reduce the risk of harmful radiation. The purpose of this review is to provide a detailed overview of various optical imaging methods that can be used for precise imaging of spinal cord injuries and their treatment. Various optical imaging techniques, including optical coherence tomography (OCT), fluorescence imaging, wearable optical technology, and neuroimaging using optical techniques, were reviewed. With OCT, surgical invasiveness is reduced and inhomogeneous structures can be captured at micrometer-level depth. Deep tissue can be imaged using fluorescence imaging. The use of wearable optical technology offers a high level of flexibility, ease of use, and precision. Using optical technologies for neuroimaging offers the comfort of being able to provide information on the structure and function of the brain simultaneously after spinal cord injury. Conventional methods, such as MRI, are not able to image precisely the spinal cord region due to the inhomogeneity in magnetic fields resulting from the highly complex anatomy of the spinal cord; however, optical technologies can accomplish this via avoiding the difficulty involved in performing imaging with high penetration depth and resolution. In addition to the above advantages, optical fiber-based techniques allow the imaging of the spinal cord to be targeted in more depth with negligible loss of light energy affecting the fiber, which ensures a high level of imaging resolution. It should also be noted that fiber Bragg gratings have also been considered to be effective devices for assessing the level of stress imposed on the spine after injury. The purpose of this review is to present recent advances and future perspectives in the field of optical devices for the diagnosis of spinal cord injuries (SCIs) and the management of the related health complications that affected individuals may experience in the future.

## 2. Optical Coherence Tomography (OCT)

**Figure 1:**
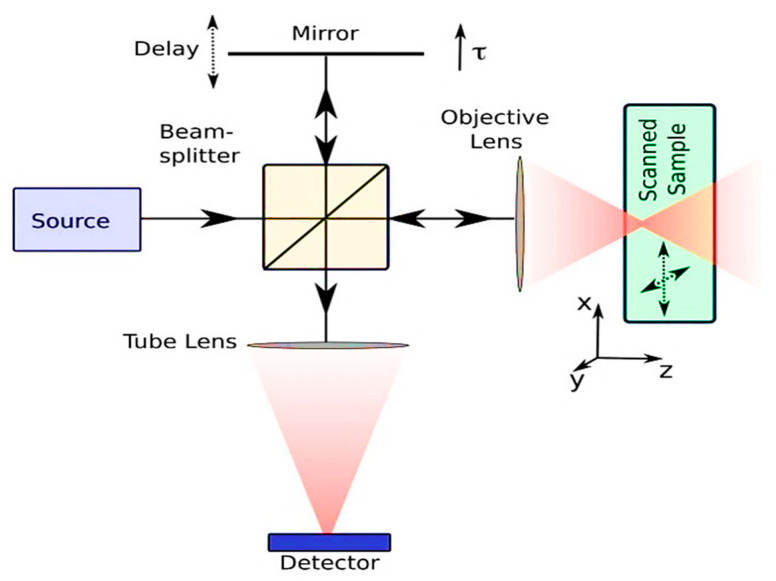
The primary working mechanism of the OCT imaging technique. Reproduced with permission from [34] (Copyright 2008, MDPI).

Navigation methods must be chosen carefully so as not to adversely affect the neurons’ functions during surgical procedures. Even though optical coherence tomography (OCT) is a relatively new imaging technique, it still provides advantages in decreasing surgical invasiveness and interference with surrounding structures. Using an interferometer, this technique of noninvasive imaging creates an image that does not require any invasive methods. Using this technology, high-resolution, cross-sectional images of inhomogeneous structures like soft biological tissues are provided on a microscopic level, as well as in 3-D. Through analyzing backscattered light reflected from tissue structures, OCT can capture structures in the deep field of view. As a result of the scattering of near-infrared (NIR) radiation, instead of its absorbtion, biological tissues can be imaged at more depth in the body than they can with other wavelengths of light. Optical coherence tomography (OCT) is a technique for creating 3-D images from backscattered NIR light emitted from a sample of interest. Since images are formed due to natural scattering, OCT is considered a noninvasive and non-disturbing technique. The biological tissues in the body possess varying optical indices and interact differently with incident light, reflecting different amplitudes. Figure 1 demonstrates the working principle of OCT. Light from a broadband optical source is incident on a beam splitter and directed towards a reference arm with a reflecting mirror and towards a sample arm mirror and a sample arm, reflected from the various tissue layers within the sample. Later, these reflected light beams recombine and interfere with each other. There is no interference between reflected optical fields when the path lengths of the reflected optical fields are shorter than the coherence length of light, provided that the path lengths of the reflected optical fields do not exceed each other, which means that interference between reflected optical fields does not occur. Compared with other optical imaging technologies, optical coherence tomography (OCT) provides several advantages, including the ability to obtain a spatial resolution of ten to fifteen meters as well as a penetration depth of three millimeters [33]. It is thought that OCT penetrates inhomogeneous tissue structures more deeply due to the use of a longer wavelength of coherent light (i.e., in the near-infrared region of the electromagnetic spectrum). In addition to high-resolution imaging capability, OCT offers numerous benefits such as fast imaging speed in the order of milliseconds, label-free imaging, cost-effectiveness, and additional information about blood flow and arrangement of tissue structures, which can be achieved using specific kinds of OCT such as Doppler OCT and birefringence OCT.

**Figure 2:**
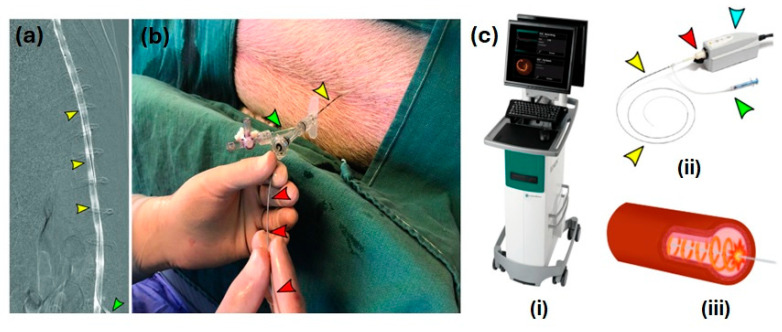
(**a**) The internal view of the lumber cistern after inserting a 16-gauge Tuohy needle into it, with yellow arrows indicating the position of the subarachnoid space visualized using a contrast agent; (**b**) the external view of inserting the OCT catheter (indicated with the red arrow) through the RHV and Tuohy needle into the lumbar cistern; (**c**) (i) the mobile unit to display captured OCT images; (ii) the OCT catheter with a large port that allows its connection to the docking system connected to the mobile unit (the blue arrow indicates the docking system and green arrow indicates the 5 mL syringe connected to catheter); and (iii) cross-sectional image of OCT probe performing a circumferential scan. Reproduced with permission from [36] (Copyright 2021, Spie. digitallibrary).

**Figure 3:**
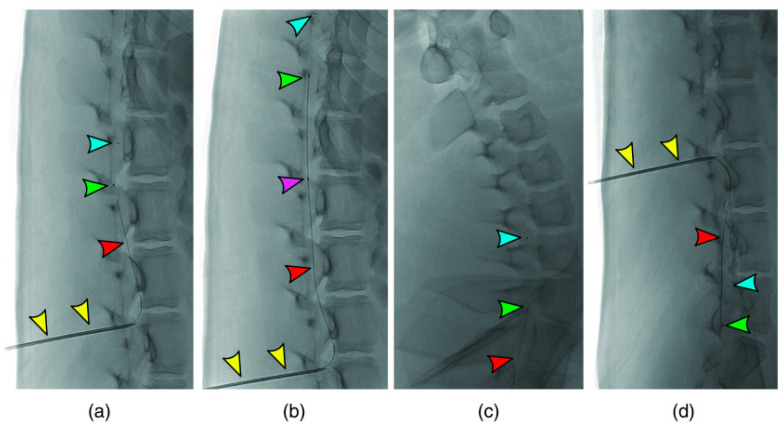
The OCT catheter’s progression into the lumber cistern through the L2/3 level (**a**,**b**). The yellow arrow indicates the Tuohy needle, blue arrow indicates the distal marker, the green arrow represents one lens marker, and the purple arrow indicates one proximal marker. The red pointer indicates the optical fiber catheter. (**c**) The catheter was seen to be advanced into the cervical spine, and (**d**) the catheter was navigated toward the sacrum (a large, triangular bone at the base of the spine) without difficulty. Reproduced with permission from [36] (Copyright 2021, Spie. digitallibrary).

In contrast to CT and MRI imaging techniques, optical coherence tomography does not require a great deal of computation to reconstruct an image [35]. As a result of the collaboration between MIT and Harvard Medical School, the first report on in vitro OCT imaging was published. In 1991, an article was published in the journal *Science* on the use of OCT to examine the peripapillary region of the retina and the coronary arteries [33]. A study published in March 2021 by Pasarikovski et al. [36], taking advantage of the advantages offered by OCT, demonstrated for the first time, to the authors’ knowledge, the use of OCT in intrathecal spinal canal imaging. The minimally invasive imaging procedure that was described in that article offers the highest spatial resolution that is currently available. A preclinical imaging study was conducted on swine and rabbits in vivo at the time of the experiment. Through the design of an OCT imaging catheter and its navigation throughout the thecal sac to visualize the spinal region, they attempted to incorporate OCT imaging with intravascular procedures. For all five animals examined, it was possible to insert an OCT catheter with a diameter of around 0.9 mm to capture cross-sectional images. To eliminate artifacts from the surrounding bone structure, the researchers investigated the possibility of obtaining images from within the thecal sac. At the same time, OCT’s high-resolution capability of 10 m can overcome the limitations in resolution that occur with CT and MRI. To facilitate the OCT catheter navigation in the spinal canal, the 16-gauge Tuohy needle was extended into the spinal canal. A contrast agent was inserted to confirm the subarachnoid positioning of the catheter (Figure 2a). In addition, Figure 2b illustrates how the OCT catheter was inserted into the spinal canal; whereas Figure 2c illustrates the arrangement of the setup components necessary for the whole OCT imaging process. Using the OCT catheter, the imaging of the cervical spine and the sacrum was performed after the catheter was advanced into the cervical spine (see Figure 3).

The various complications caused by SCI have been the subject of numerous studies conducted by multiple groups worldwide. Surgical procedures involving invasive surgery, which are often adopted, can result in a variety of health-related conditions, owing to their invasive nature. The proposed method eliminates the need for open surgery that involves using an OCT catheter to intervene directly in the thecal sac, with its obvious risks. Instead, a 16 gauge Tuohy needle is applied directly to the thecal sac. Through using this technique instead of putting catheters inside the body for several days, the chances of an infection are minimized compared with older techniques. In terms of neurological conditions of the subject, the presented approach with an OCT catheter of 0.9 mm diameter is considered to be safe.

**Figure 4:**
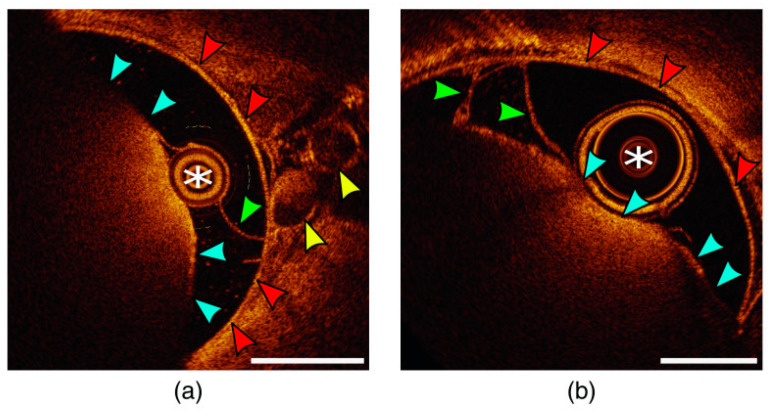
Intrathecal cervical spine canal OCT imaging: (**a**,**b**) a white asterisk represents the location within the subarachnoid space, blue arrows represent the pile lining of the spinal cord, green arrows represent the arachnoid bands, and a lateral dentate ligament, yellow arrows represent the epidural veins and red arrow indicates the dura. White bars = 1 mm. Reproduced with permission from [36] (Copyright 2021, Spie. digitallibrary).

Anatomically standard spinal cords of swine and rabbits are used for this procedure as they are anatomically standard. Figure 4 provides an image of the intrathecal cervical spine showing a clear visualization of the dura, the subarachnoid space, the epidural vessels, the dentate ligaments, the nerve roots, and the rootlets of the spinal cord.

With the use of OCT, these images can be visualized in real time, which can help the clinician to see the places where there is spinal cord injury, such as tumors, cysts, vascular malformations, and so on. OCT is a very useful tool for obtaining a detailed image of the whole spinal cord; however, it may have limitations due to its capability to penetrate to a depth of 3 mm. Ding Z. et al. [37] in 2016 investigated the practicability of polarization-sensitive (PS) OCT for imaging different spinal structures for smooth interventional procedures, because conventional intensity-based OCT was found unable to provide tissue-specific contrast, making it difficult to distinguish between tissues [38,39]. As a functional extension of optical coherence tomography (OCT), polarization-sensitive optical coherence tomography (PS-OCT) exploits light polarization to enhance the contrast provided in images created through polarization-sensitive OCT [40,41]. As a result of the ability of various tissues to alter their polarization of reflected or scattered light, PS-OCT can be used to provide morphological information via adding contrast to reflected or scattered light [42]. Phase retardation and optic axis orientation are two polarization parameters that can modify the polarization state of reflected or scattered light at the time of reflection or scattering. Light’s polarization property can be used to organize biological tissues on a nanoscale with a high degree of distinguishability.

Reseachers investigated paraspinal structures including subcutaneous fat, supraspinous ligament, interspinous ligament, dura, and spinal cord in both ex vivo porcine models and in vivo piglet models using a PS-OCT system (PSOCT1300, Thorlabs Inc.). A commercially available PS-OCT system was used as the imaging instrument. It was possible to represent both intensity contrast images and polarization contrast images simultaneously in both approaches.

## 3. Fluorescence Imaging

The use of fluorescent microscopy has been developed for observing deep tissues. A confocal microscope gives images that are in focus all the time and eliminates out-of-focus details in thick samples [43]. A multiphoton excitation technique was first demonstrated in 2014 [44] and is the most recent innovation in this field. With the help of two-photon stimulated fluorescence imaging and Hoechst 33,258 stain, researchers produced an optical slice of live, cultured pig kidney cells. Instead of using visible light to activate chromophores, it may be possible to use infrared lasers to activate chromophores with two photons instead of visible light. This avoids typical photodynamic disturbance and enables prolonged fluorescence investigations of living cells. Victria et al. discussed the benefits of multiphoton excitation techniques for observing deeper tissue through confocal microscopy imaging [45], such as (i) adopting a longer wavelength of light to allow deeper penetration; (ii) compared with confocal microscopy, multiphoton imaging employs longer wavelengths of light, such as infrared light (700–1000 nm), which has significantly less energy and hence produces much less photodamage or phototoxicity to cells and tissues; (iii) long-term studies of cells and molecules located deep within live tissues are made possible by multiphoton microscopy; (iv) unlike single-photon confocal microscopy, multiphoton microscopy minimizes photobleaching outside of the focal plane since excitation and emission occur only at the focal plane. A multiphoton microscope produces fluorescence of the same intensity as single-photon microscopy when it uses low-energy photons. There is a need to maintain the development of therapeutic modules to treat spinal cord injuries caused by degeneration processes. There is a tendency for spinal cord tissue to degenerate in the aftermath of traumatic injury, as a result of central damage such as hemorrhaging and axonal shredding. It has been suggested that secondary degeneration can be caused by ischemia, tissue edema, glutamate-induced excitotoxicity, or a rise in intracellular calcium levels. Degeneration of these cells is also influenced by apoptosis, which is a process of cell death. There are two main parts that make up the structure of the spinal cord: gray matter, which contains the neuronal cell bodies, and white matter, which primarily contains the axons and myelin. One of the most important organs implicated in the initiation of paresis is the pyramidal pathway that connects the central motor neurons in the cerebral cortex with the spinal motor neurons. Due to this, it is crucial to monitor the axon degeneration processes in the dorsal funiculi in the wake of spinal cord injury. In an earlier study, Horiuchi et al. pioneered the use of a multiphoton-stimulated fluorescence microscope using an Ytterbium (Yb)-fiber laser at 1045 nm for in vivo imaging of axon fibers in the dorsal funiculi. Furthermore, recent studies have shown that multi-colored fluorescent proteins can easily be expressed after genome editing in nerve cells to analyze their structure, metabolism, and intercellular connections in real time [46].

**Figure 5:**
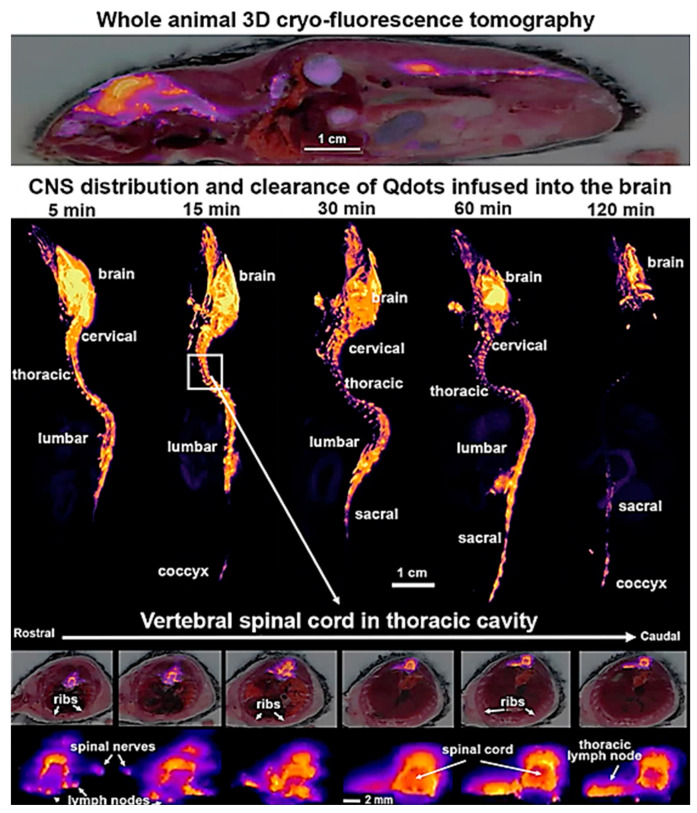
White light and cryo-fluorescence tomography (CFT) at the level of the vertebral spinal cord post-infusion of Qdots ICV. Reproduced with permission from [50] (Copyright 2023, MDPI).

Fluorescent peptide was produced by Nguyen et al. to highlight peripheral nerves during surgery on mice. Because of the blood–brain barrier (BBB), these fluorescent probes cannot penetrate the central nervous system (CNS), including the brain and spinal cord. Furthermore, Gibbs-Strauss et al. reported imaging of the trigeminal ganglia, dorsal root ganglia, and intervertebral disk. In this context, these fluorescent agents could not highlight the central nervous system because of their size [47]. Nevertheless, fluorescence-guided surgery (FGS) has been identified as a potential means of improving surgical accuracy and visibility [48]. (E, E)-1,4-bis(p-aminoethyl)-2-methoxy benzene (BMB), an efficient and rapid nerve-highlighting fluorophore, was administered epidurally in rabbits by Liu et al. [49]. Stokes et al. examined the movement of tracers from mice’s spinal cords through quantum dots (QdotR 605 ITKTM amino (PEG)) using cryo-fluorescence tomography (CFT) (Figure 5) [50].

## 4. Wearable Optical Technology

**Figure 6:**
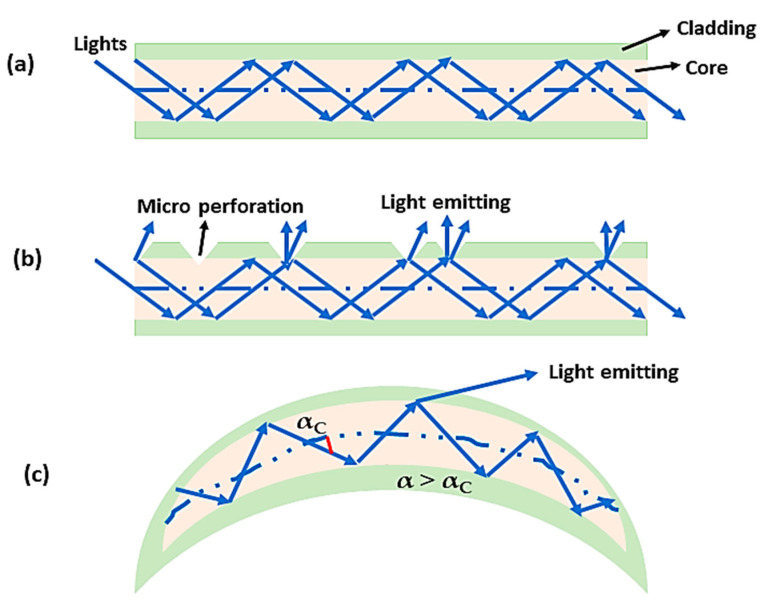
Principles of light emission for wearable optical technology: (**a**) conventional optical fiber; (**b**) cladding perforation; (**c**) a macro-bent optical fiber.

A wearable optical device that measures a variety of changes, such as chemical, physical, and biological variations, can be utilized to assess a variety of changes using wavelength, phase, and intensity variations. Modern medicine has had a significant impact on how light and optical techniques are applied in clinical practice to treat disease. These techniques have proven to be very effective [51]. Many different wavelengths of light can be used in light therapy. It is frequently used for metabolic disorders, tendon injuries, pain relief, healing of tissues, etc. A variety of fiber optics with superior optical and thermal properties for light therapy, developed in conjunction with textile materials and worn near the skin, have been combined. In this context, choosing a wavelength of light that penetrates human tissues at a certain depth is one of the most important factors when it comes to light treatment because these wavelengths have different therapeutic effects depending on the depth at which they penetrate. The ability of near-infrared light to affect chromatophores and to increase the synthesis of ATP (adenosine triphosphate) in mitochondria can accelerate wound healing. Wearable devices that can be worn on the body and can be used for various purposes have recently been equipped with optics made from optical fibers. In response to the requirement for distinctive clothing in vibrant colors, optical fibers have been successfully incorporated into fabrics for illumination to achieve this objective [52]. In contrast to conventional optical fibers used for signal transmission, whereby light is reflected inside the fiber and released at its end, as shown in Figure 6a, optical fibers designed for wearable illuminated clothing feature micro-perforations on the lateral side that allow light to pass through the cladding and into the core (Figure 6b). When the light scatters at the holes in the fiber, this causes light to leak through the fiber surface as a result of light emission on the surface of the fiber [53]. An alternate method of achieving light emission is to bend the optical fiber macroscopically (Figure 6c) until the light propagation angle (α) exceeds the critical angle (αC), causing a portion of the light to be emitted from the optic [52].

It is of utmost importance for physicians to evaluate the patient’s postoperative outcome regularly after spinal surgery to guide their decision making. There is no doubt that this is crucial for the patient’s health and for assessing how effective the surgical intervention has been on their health. There are several types of patient-reported outcome measures (PROMs) that for many years have been used to assess long-term outcomes following surgery. These include the 36-item Short-Form Health Questionnaire (SF-36), the Oswestry Disability Index (ODI), and the EuroQol (Eq-5d) [54]. Despite the benefits of PROMs in terms of providing direct information from the patient, one significant drawback is the fact that the responses are subject to bias as a result of the subjective nature of the questionnaire [55]. A study was conducted by Pryce and associates who evaluated patients who had undergone surgery for lumbar spinal stenosis (LSS). To determine patients’ functional status, the ODI, SF-36, and levels of physical activity were used to track their exercise intensity and duration. The SF-36’s physical function subscale was the best outcome measure, but its average correlation coefficient (r = 0.53) did not predict patients’ levels of physical activity. There is some evidence that subjective pain assessments and physical activity assessments are not always correlated with actual physical activity, as reported in that study [56].

**Figure 7:**
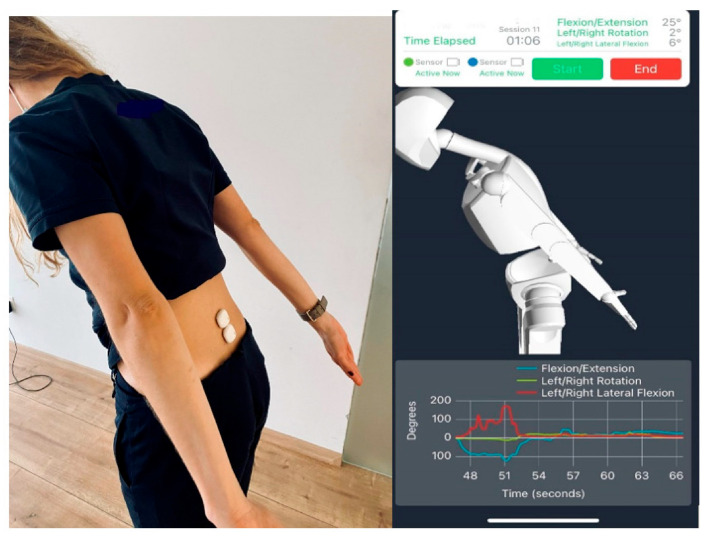
Application of wearable sensors in the lumbar spine. Reproducing software and recorded kinematic data. Reproduced with permission from [59] (Copyright 2022, MDPI).

An important use of wearable devices for spinal surgery is to accurately measure a patient’s daily step count, which is one of the most promising uses of wearables available on the market today [57]. In the past, pedometers have been used to keep track of how many steps are taken each day. Early models of pedometers used mechanical switches as the means of counting steps. In the past ten to fifteen years, accelerometer-based pedometers have gained popularity due to their ability to measure additional step metrics, like cadence, as well as providing a more accurate step count. It has been suggested that accelerometer-based movement tracking may be helpful in clinical practice since walking ability and physical activity are significant indicators of overall health and outcome measures [58]. As part of preventative healthcare, wearable systems have also been created to monitor the lumbar spine’s motion, curvature, and alignment, emphasizing the prospect of preventative care. We need to bend our spine forward or flex our spine when performing daily chores, but this may be highly stressful on the lumbar spine and result in disc distortion (Figure 7).

According to Tsuchiya et al., [60] a wearable sensor system has been developed that is capable of detecting postures that are associated with an increased risk of back pain. Using the device, one can estimate daily lumbar load as well as lumbosacral alignment. As part of the system, accelerators and flex sensors are integrated with compression sportswear, making up a complete system. With the help of X-ray imaging of the lumbosacral spine, the best sensor combinations for the suit were identified. There is still a need to carry out more work to increase the precision of the lumbosacral alignment estimates, and the researchers acknowledged that different methods of correction were responsible for estimation error. In addition, estimations of lumbar loading will have to be made with more accuracy [60].

## 5. Neuroimaging with Optical Techniques

In recent years, it has become clear that neuroimaging techniques have become an effective means for the diagnosis of a wide range of neurological disorders because of their ability to provide information on both structural as well as functional aspects of disease. In addition to using conventional X-rays, computed tomography, magnetic resonance imaging, positron emission tomography, magnetoencephalography (MEG), electroencephalography (EEG), and combinations of these former techniques, neuroscientists and clinicians can gain a deeper understanding of the brain’s structural and functional pathology with these new imaging techniques. Neuroimaging techniques such as diffuse optical imaging, near-infrared spectroscopy, and cranial ultrasound have recently been developed as additional neuroimaging techniques. It is an intrinsic property of the spine to limit the spinal cord’s accessibility. Because of this, it is difficult to use neuroimaging techniques to evaluate the impact of traumatic diseases or injuries and to understand the functioning of the spinal cord in the human body. High-contrast pictures of soft tissues and bony structures can be obtained with conventional plain radiography and computed tomography (CT). These imaging modalities are typically utilized in orthopedics to visualize spinal structures [61]. Several factors make the spine one of the most difficult body structures to image with MRI. These factors include the small cross-sectional dimensions of the spinal cord, the relatively large physiological movement of the spinal cord and its adjacent tissues during the cardiac and respiratory cycles, and the inhomogeneity of magnetic fields created by the interfaces between the surrounding bone, ligaments, and other soft tissues, as well as the cerebrospinal fluid inside the spinal canal. It is particularly challenging for patients with metallic implant or equipment artifacts to undergo such imaging. This can often pose a challenge for them because metallic artifacts can be caused [62]. It has been demonstrated that conventional MRI techniques are capable of producing precise, high-resolution images of the spinal cord in a highly accurate way. Because of continuous advances in imaging sequences, conventional MRIs have become widely used as an essential imaging modality for most spinal illnesses. Nevertheless, since changes in signal strength in various spinal cord diseases are usually non-specific and weakly linked to neurological and functional impairment, conventional MRI is not a good indicator of the intrinsic integrity and physiological state of spinal cord tissue since changes in signal strength are generally non-specific and weakly correlated with neurological and functional impairment. Therefore, they are not able to provide prognostic information that is particularly reliable at the microstructural and operational levels [63]. With the use of optical imaging techniques, it has been possible to study the structural and functional connections in rodent brains. In the study of cellular and vascular architecture, neural activity, blood flow, blood pressure, and oxygen saturation, these methods have allowed researchers to examine cellular and vascular architecture. Two or more photon excitations and calcium-sensitive dye imaging (CaSDI) have been extensively employed in optical neuroimaging techniques [64,65,66], as have voltage-sensitive dye imaging (VSDI) [67,68], laser speckle contrast imaging (LSCI) [69,70,71], optical intrinsic signal imaging (OISI) [72,73,74], and ultrasound imaging (USI) combined with light [75,76], all of which are optical neuroimaging techniques. Below, we introduce some of the distinctive characteristics of neuroimaging technologies that can be found on the market today.

(a) The 2PE imaging method is a nonlinear laser-scanning fluorescence microscopy technique with sub-micrometer spatial resolution and 500 μm~1 mm depth of view, and has become more popular in neuroscience. It has been demonstrated that near-infrared two-photon excitation wavelengths can be used as a stimulus for structural fluorescence imaging as well as for CaSDI and VSDI for the imaging of neural activity in a wide field of view. Its wavelength is about twice as long as conventional wavelengths used for confocal or epifluorescence excitation [77,78,79]. Due to recent advances in MPE, especially 3PE, it is now possible to obtain even deeper functional imaging below a depth of 1 mm.
(b) Using OISI with spatial and temporal resolutions of up to 100 mm and 2 s, we can visualize local microcirculation and cortical functional architecture label-free, with resolutions of up to 100 mm and 2 s, respectively. Earlier studies used this technique to examine the functional connections in the large-area cortex of mice, as well as to examine the functional connections on the exposed mouse skulls [80,81,82]. It has been shown that the use of multicolored light illumination can be used to assess the concentrations of deoxy- and oxyhemoglobin in different areas of the brain [83,84,85]. It has been used for a long time to infer the activity of the brain, based on changes in cortical reflectance caused by hemodynamic responses, which can be measured by OISI.
(c) A laser speckle is a random interference pattern that appears when coherent light is scattered within tissue, often referred to as a laser beam. A dynamic movement within the live tissue generates changes in the speckle pattern as a result of the dynamic movement of scatterers, such as red blood cells, within the tissue. Using this technique called laser speckle color imaging (LSCI), we can produce a two-dimensional representation of tissue perfusion or blood flow in a two-dimensional space. An LSCI system is capable of achieving a time resolution of 10 msec to 10 sec as well as a spatial resolution of 10 μm, depending on the application. As a result of its shallow light-penetration depth, this technology can provide only a superficial blood flow map [86,87].
(d) CaSDI and VSDI can be used simultaneously to measure the activity of several categories of neurons at the same time, since these methods provide temporal and spatial resolutions in the msec and μm ranges, respectively. In these optical neuronal imaging methods, certain dyes that are sensitive to neural activity are used to detect neural activity. Depending on the type of measurement, these methods can be used to monitor cellular or sub-cellular neural activity using dyes that glow when an action potential is generated [88,89].

## 6. SCI Treatment with Optical Fiber-Based Devices

There has already been discussion of how SCIs can result in impairments in the sensory, motor, and autonomic functions of the body. An injury to the spine may result in a fracture in the spinal column, which can result in the loss of neurological function as a result of the injury. Additionally, non-traumatic SCIs may lead to the development of infections, degenerative disc disease, and tumors as a result of injury [90,91,92]. It is highly desirable to develop a therapeutic approach that can repair the effects of SCIs as well as cure it in the context of all these facts [91,93,94]. Low-level laser therapy, also known as photo biomodulation, is considered a potential non-invasive therapeutic approach to cure spinal cord injuries by effectively treating inflammation through the reduction of inflammation [95]. There is strong evidence that this therapeutic approach is effective in repairing neuronal tissue and minimizing injury complications and that irradiating SCI sites with near-infrared light in a near-infrared wavelength of 810 nm, a power of 150 mW, and a dosing rate of 1500 J/cm^2^ has the potential to reduce the immunoinflammatory response, promote neuronal survival, and improve locomotor function in patients with SCIs.

**Figure 8:**
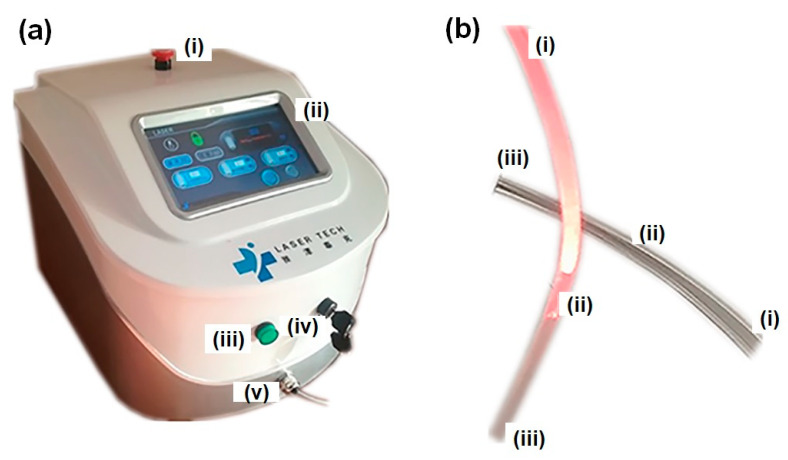
(**a**) Therapeutic semiconductor laser device with constant 810 nm wavelength: (i) button for emergency shutdown; (ii) interface for digital control; (iii) functional status indication lamp; (iv) power button; (v) interface for optical fibers. (**b**) (i–ii) light-emitting portion of an optical fiber; (ii–iii) remaining optical fiber components. Reproduced with permission from [96] (Copyright 2020, Wiley).

**Figure 9:**
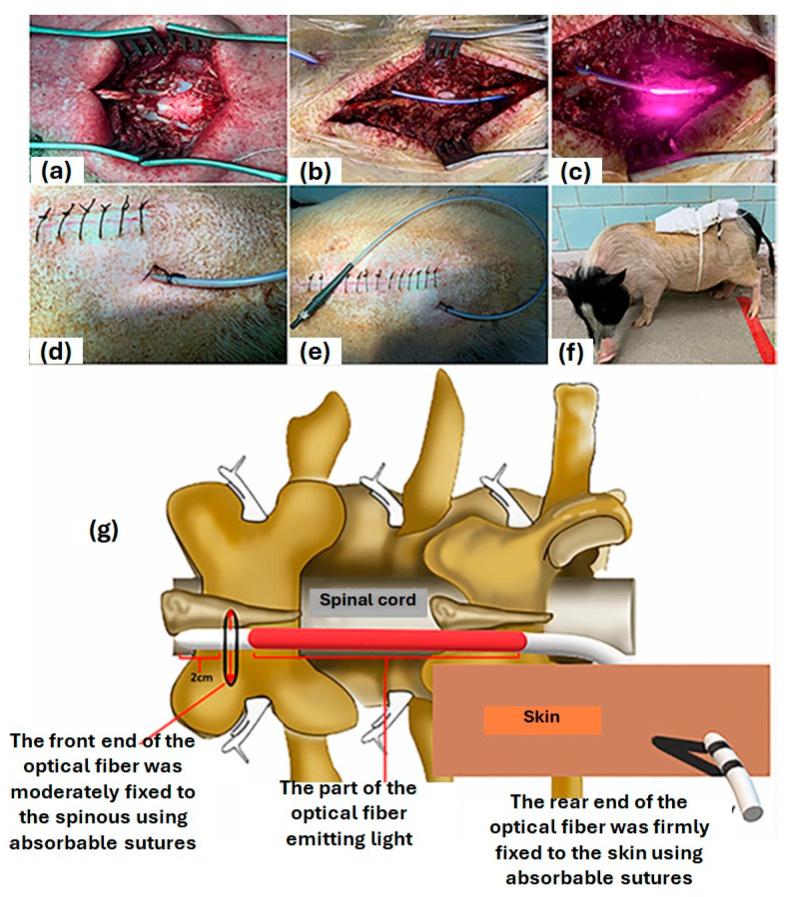
(**a**) The exposed T9 spinal cord; (**b**) the front end of the optical fibre was fixed to the spinous using absorbable sutures; (**c**) projection of light energy directly on the spinal cord surface; (**d**,**e**) the optical fibre was fixed tightly around the skin with absorbable suture; (**f**) the pig retained its motor ability after the operation; (**g**) schematic representation of implantation, fixation and irradiation using the optical fiber. Reproduced with permission from [96] (Copyright 2020, Wiley).

Laser therapy is one of the most efficient methods of curing SCIs but it suffers from substantial losses of light energy due to indirect irradiation to the injured area, limiting its effectiveness. This loss of light energy must be addressed when the light needs to reach into the deeper spinal cord tissue to perform an effective restorative procedure. To overcome this situation, it is necessary to design a laser therapy device that directly radiates light energy onto the surface of the spinal cord to be able to provide laser therapy. In a piglet model, Liang et al., 2019, relied on the fact that there is a similarity between the spinal structure of Bama miniature pigs and humans and carried out laser therapy (photo biomodulation) to treat spinal cord injuries [96], where optical fiber was diffused on the spinal cords of piglets. The main component of the designed device was an 810 nm continuous-wavelength semiconductor laser, equipped with all the necessary controls, as well as a high-transparency cylindrical silica-coated medical optical fiber with a core diameter of 600 m and an outer diameter of 300 m. Due to its flexibility and biocompatibility, the optical fiber did not affect the camera’s optical properties in any way (Figure 8). As soon as the spine was exposed following a surgical procedure, the washed front and rear ends of the optical fiber were moderately fixed to the soft tissues of the spinal cord and the skin, respectively, using absorbable sutures. In addition to the optical fiber’s back end, the laser irradiation device was interfaced to the back end of the fiber in order to check the fiber’s light emission properties and ensure that the light could be delivered to the spinal cord’s surface (Figure 9).

**Figure 10:**
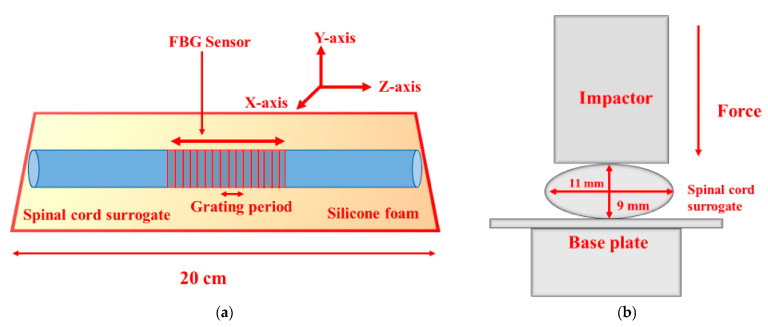
(**a**) An illustration of the longitudinal mid-cross-section of an instrumented spinal cord surrogate that incorporates an integrated fiber Bragg grating (FBG) sensor; (**b**) transverse view of the mechanical and optical setup of the instrumented spinal cord surrogate. Reproduced with permission from [110] (Copyright 2021, MDPI).

The positioning of the optical fibers on the spinal cord tissue was investigated using 3.0 T MR imaging to determine whether the fibers were causing any compression on its tissues, and it was discovered that they were positioned at a certain distance from the surface of the spinal cord to avoid any obstructions from influencing the communication. With the use of optical fiber implantation, therapy using lasers can provide a new tool for the treatment of spinal cord injury. To maintain patients’ spinal health after traumatic spinal injury, it is crucial to understand the real-time biomechanics of soft tissues, and in vitro models can aid in understanding these processes. It is vital to know the exact degree of compression of the spinal cord following a traumatic injury, as severe compression can result. Several current sensing techniques can provide information regarding this stressful situation that the spinal cord experiences. The development of an optical pressure-sensing system based on a fiber Bragg grating and a narrow-band optical filter was conducted to identify and display in real time the transverse compression inside a spinal cord surrogate. In recent years, many factors, such as temperature, pressure, displacement, humidity, strain, variation, and so on, have been studied using fiber Bragg gratings (FBGs) as sensors, to obtain more information about them [97,98,99,100,101,102,103,104,105,106,107]. During the fabrication of FBGs, a periodic structure is fabricated along the core of the optical fiber to provide the necessary energy coupling between the forward and backward propagating modes of the fiber. It is possible to transfer energy between the forward propagating mode and the backward propagating mode in an FBG written in single-mode fiber, once two identical backward propagating modes are coupled. It is for this reason that an FBG serves as a bandpass optical filter in the sense that it reflects specific wavelengths [108]. This model enabled a susceptible strain measurement at a cm-scale axial resolution inside a surrogate spinal cord with a significantly fast response time of only 20 s, comparable to those achieved in real-time in vitro studies on traumatic spinal cord injuries. The 3D-printing method of casting silicone elastomer foam has been used in the preparation of spinal cord surrogates [109]. To achieve this, the foam was formulated in the form of an ellipsoidal shape with measurements imitating those of the transverse section of the human thoracic spinal cord (Figure 10).

**Figure 11:**
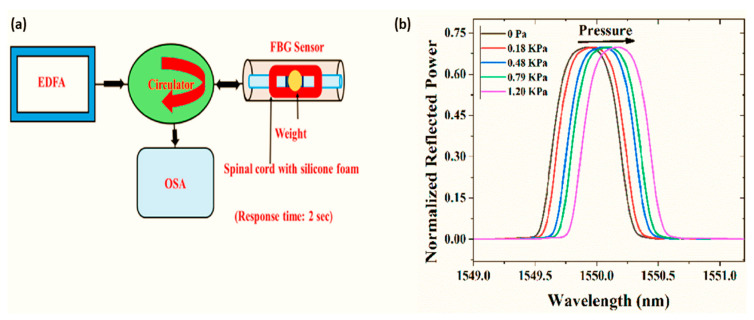
(**a**) An experimental setup for the characterization of a fiber Bragg grating (FBG) sensor and (**b**) the sensor’s reflection spectra (normalized with input power) for different transverse pressures on the spinal cord surrogate, obtained using the optical spectrum analyzer. Reproduced with permission from [110] (Copyright 2021, MDPI).

The picture shows a simple optical FBG sensor winding around a spinal cord sensor system. The researchers recorded data that indicated the variation of wavelengths along with the transmitted power for different pressure levels. They were able to measure the pressure change around the spinal cord by changing the transmitted power and in this way, they were able to determine the characteristics of the sensor (see Figure 11).

## 7. Photoacoustic Imaging through Plasmonic Nanoparticle

**Figure 12:**
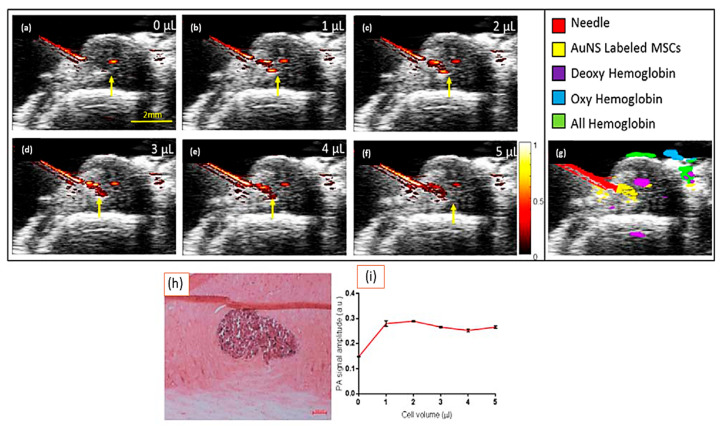
Using US and PA imaging techniques, researchers imaged an injection of AuNS-labeled MSCs into the spinal cord of a rat. (**a**–**f**) We were able to capture ultrasound and photoacoustic images in the same sequence after injecting 0, 1, 2, 3, 4, and 5 L of AuNS-labeled MSCs using the same protocol. A scale bar measuring 2 mm is shown in the figure; (**g**) the distribution of all photoacoustic absorbers after injecting 5L of AuNS-labeled MSCs into the spinal cord; (**h**) a photomicrograph showing transplanted AuNS-labeled MSCs in the gray matter of the spinal cord, with a scale bar of 2 mm; (**i**) a graph of the photoacoustic signal with an average amplitude of 700 nm throughout the experiment. Error bars are used to indicate the standard deviation of the data. Yellow arrows represent the needle shaft. Reproduced with permission from [129] (Copyright 2018, American Chemical Society).

The diseases and disorders of the spinal cord that tend to have a terrible prognosis are often those that are caused by neurodegenerative illnesses or accidents, such as multiple sclerosis, spinal cord injury, and amyotrophic lateral sclerosis (ALS) [111], a condition in which spinal cord cells degenerate and die. One of the most appealing treatment modalities for these neurodegenerative diseases is stem cell transplantation, and several modalities are being researched. As far as research in stem cell therapy is concerned, it continues to be one of the most active areas in the field as it relates to clinical trials. Several stem cell-based clinical studies are currently being conducted to investigate the effectiveness of stem cells in treating a wide range of illnesses, such as cancer, diabetes, osteoarthritis, and neurological diseases. Specifically, mesenchymal stem cells (MSCs) are multipotent stem cells that can be derived from adult stem cells and can differentiate into a wide range of lineages, including osteoblasts, adipocytes, and chondrocytes [112]. The use of MSCs has been applied in more than 800 clinical trials, of which more than 40 have involved the application of the cells for spinal cord regeneration. There are three main obstacles that need to be overcome if spinal cord stem cell transplantation is to be more effective. These obstacles include (i) precise real-time delivery of stem cells to desired locations, (ii) non-invasive, long-term in vivo cell monitoring, and (iii) the evaluation of stem cell survival over time following the delivery of stem cells. As the delicate tissue surrounding the spinal cord is extremely susceptible to direct manipulation, it is highly recommended that therapeutics be administered with extreme caution and precision. There is a possibility that the white matter pathways in the spinal cord may be damaged if the needle moves abruptly or incorrectly, which could have a detrimental effect on the patient’s health. It is necessary to have an automated, safe, and cost-effective method that is compatible with operating rooms to perform and accomplish image-guided cell distribution within the spinal cord in real time. Researchers have shown, through a series of research studies, that direct injections of drugs into the tissues of the spinal cord are more beneficial to the treatment of spinal cord injury than systemic administration of drugs [113]. Novel techniques have been devised to enhance the procedure’s safety and effectiveness, owing to the delicate nature and elevated danger of direct spinal cord injection [114,115]. Taking a clinical perspective, real-time imaging guiding systems make it possible to deliver interventions to patients more precisely, allowing medical professionals to monitor and adjust treatment in the operating room in real time, thereby increasing the chances of a successful outcome. As mentioned above, the accuracy of the injection’s targeting is essential to the success of the treatment, which largely depends on accurately accessing the location(s) that have to be reached during the injection [116]. The use of traditional preoperative imaging techniques such as magnetic resonance imaging (MRI) can determine the exact injection coordinates for a procedure in advance. This is, however, far from being a perfect solution. In fact, there is a possibility that the intended course, as evidenced by preoperative imaging, may not work when surgery is carried out. It has been found that a variety of techniques, including bioluminescence, optical imaging, positron emission tomography, and magnetic resonance imaging, can be used to label and image transplanted cells in vivo as viable approaches for this purpose [117,118,119]. Despite their positive findings, real-time feedback is a difficult task when it comes to these technologies because of their high cost, the radiation dose required for PET imaging, and the difficulty of using them intraoperatively, despite the positive findings relating to their use. With ultrasound-guided photoacoustic imaging (PA), which is a relatively new imaging modality able to guide direct stem cell distribution to the spinal cord as well as image the labeled stem cells once they have been injected into the spinal cord, it is possible to direct stem cell distribution to the spinal cord. A nanosecond-pulsed laser is used in PA imaging in order to exploit the photoacoustic phenomenon that occurs when light is converted into sound. During the absorption of electromagnetic radiation by optical absorbers, a pressure wave is created as a result of the induced localized thermal deposition. As optical absorption, not scattering, is the primary source of contrast in three-dimensional optical systems, it is possible to achieve highly specific optical contrast at depths of several centimeters. Several similar hardware components, including modified ultrasound imaging (US imaging) transducers, can be used in this process because it resembles US imaging in many ways. Moreover, US imaging offers insightful anatomical data to enhance the functional data of PA imaging. Combined ultrasound/photoacoustic (US/PA) imaging is an appealing option for a variety of clinical applications due to its small size, mobility, lack of radiation exposure, familiarity to clinical staff, and accessibility [120,121]. A large range of dyes and nanoparticles can be used as contrast agents for PA imaging [122]. Plasmonic gold nanospheres (AuNSs) are promising contrast agents for labeling stem cells for PA image-guided administration [123,124,125]. In vivo labeling and imaging of MSCs has shown that this method represents a highly promising application of this photoacoustic contrast agent [123,124]. Incubation of MSCs with citrate-stabilized AuNSs precedes transplantation. MSCs become optically active for PA imaging when they effectively absorb these AuNSs, without the need for extra processing. As examined in vitro, AuNSs have no detrimental impact on cell division, proliferation, or therapeutic potential [126,127,128]. The work of Eleanor et al. has demonstrated that by utilizing US/PA imaging along with gold nanospheres (AuNSs) nanoparticles, it is possible to direct the injection of AuNS-labeled MSCs to specific anatomical targets within the spinal cord and to track the injection of the MSCs as they enter the cord (Figure 12). Using an eosin-stained transverse section of the spinal cord, the researchers were able to see the bolus of transplanted cells within the grey matter of the cord, proving that the cells were successfully delivered to the ventral grey matter of the cord and that the spectroscopic analysis was accurate (Figure 12h). A two-dimensional transverse cross-section showing the average amplitude of the PA signal was examined for ten frames for every injection volume (Figure 12i).

Table 1 represents the critical parameters of all the discussed optical devices for SCI diagnosis and management.

## 8. Future Perspectives and Conclusions

Discussing all these devices involving optical methods can identify suitable ways to deal with the highly complex spinal cord anatomy for precise imaging, which could aid in effective diagnosis and management of patients suffering from SCIs. Conventional methods have contributed significantly over the years, but there is room for further improvement in treatment methods from a future perspective. All of the optical methods discussed pave the way to achieve high-resolution spinal cord imaging non-invasively compared with conventional methods. Despite offering various advantages, optical methods also have their own limitations, which must be addressed. In the future, these optical techniques aim to improve image resolution to the maximum extent possible with targeted imaging of the core of the spinal cord. It is difficult to reach the spinal cord’s core because of its complex architecture. Techniques like optogenetics are nowadays attracting great attention from researchers, as this technique stimulates the spinal cord neurons and helps treat SCIs. Optical tools such as optical fiber significantly strengthen this imaging method because they can be flexible, slim, and have navigation capability through narrow regions [130]. Furthermore, the main obstacle in securing high-resolution images of the closest region of the spinal cord using optical methods is the scattering of light energy from tissues around the spinal cord. The methods to be investigated require accessing the spinal cord to its maximum extent without disturbing its surroundings, so that surgical complications can be avoided. Given this, the optical tissue clearing method has emerged to improve tissue transparency and reduce the light-scattering effect, with the aim of visualizing the spinal cord accurately for better treatment planning [131]. Another potential imaging method for SCIs is the terahertz imaging method, which is currently in its development phase yet attracting colossal interest from researchers in dealing with SCI diagnosis, and the treatment and recovery of individuals with SCIs. Various biological tissues are susceptible to terahertz absorption, which certainly provides this technique with the capability of specific tissue identification with deeper penetration through the human body, helping to understand injury better and non-invasively [132]. In the era of artificial intelligence (AI), the collaboration of AI and optical imaging methods may improve the diagnosis process. AI can help provide a comprehensive analysis of obtained imaging data with other clinical information. This collaboration of AI and optical methods can strengthen prognosis and enhance the quality of life of individuals suffering from SCIs [133]. As an advancement in SCI diagnosis, treatment, and recovery, better and more accessible wearable and consumer-grade technologies are urgently needed to improve the quality of life for individuals with SCIs. Post-SCI suffering, such as pressure ulcers in the affected subject, can be severe and require great attention. In this regard, optical fiber Bragg grating (FBG)-based optical sensors may play a vital role. FBG can monitor pressure variation for wheelchair users and prevent ulceration. The FBG technique provides the capability of multiplexing, immunity to electromagnetic interference, capability to withstand humidity, and compactness in size [134].

In conclusion, spinal health is crucial as it regulates human sensory and motor functions. External events such as accidents, falls, and collisions and internal causes such as tumors, infections, insufficient blood flow, and degenerative diseases may cause severe SCIs. SCIs affect human life emotionally, physically, and financially. The expected cost of medical treatment for SCIs is appreciably high globally. Hence, adopting preventive measures may subdue the consequences. The existing approaches, such as stem cell therapy, neuroprotective drugs, and electrical stimulation, offer improvement in the functional outcomes for patients with SCIs in terms of tissue regeneration, minimization of secondary damage, and enhancement of motor function to improve quality of life. However, these approaches are experimental and need clinical trials. Given all this, processes that can provide reconstruction to revive the motor and sensory functions and supportive care to handle the consequences of SCIs are required. Because of this, precise early-stage diagnosis of SCIs is vital, and imaging technologies could play an important role. It is crucial to provide a clear path for surgical tools to the target sites without hindering the complex anatomy of the spinal cord when performing surgical procedures. Conventional methods such as endoscopy use harmful radiation, and the radiation-less techniques of MRI, ultrasound, and diffusion tensor imaging (DTI) suffer from substandard image resolution, poor tissue specificity, expensiveness, and cumbersomeness. Surgical tools for SCIs must be compatible with the surgical procedures used in these imaging techniques. Given all these pressing concerns, optical imaging techniques such as OCT, fluorescence imaging, wearable optical techniques, neuroimaging with optical methods, optical fiber-based methods, and photoacoustic imaging through plasmonic nanoparticles contribute significantly to SCI treatment and repairs. All of these mentioned optical techniques have been discussed in detail and found to offer numerous benefits including non-invasiveness, high-resolution imaging of target sites without disturbing the anatomy of the spinal cord, tissue differentiability with depth resolution, online monitoring capability, and postinjury repair.

## Figures and Tables

**Table 1:** Highlighting the critical parameters of discussed optical imaging methods.

S.No.	Methods	Critical Parameters	References
1	Optical coherence tomography (OCT)	Spatial resolution of about 10 to 15 μm and penetration depth of about 3 mm.	[33]
2	Fluorescence imaging	Capability to highlight deep tissue.	[48,49,50]
3	Wearable optical technology	Flexible, easy to wear, precision.	[54]
4	Neuroimaging with optical techniques	Sub-micrometer spatial resolution and 500 μm~1 mm depth of view (2PE), spatial and temporal resolutions of around 100 μm and 2 s, respectively (OISI), time resolution of 10 msec to 10 sec and a spatial resolution of 10 μm (LSCI).	[72,73,74,76,77,78,81,82]
5	SCI treatment with optical fiber-based devices	Flexibility, biocompatibility, minimal loss of light energy, and ability to measure the stress on the spinal cord post-injury.	[93,94]
6	Photoacoustic imaging through plasmonic nanoparticles	Radiationless targeted imaging.	[124]
